# Miscellany

**Published:** 1893-12

**Authors:** 


### Miscellany.

The Health Commissioner of Buffalo, Dr. Ernest Wende, has issued the following circular to the members of the medical profession :

Hereafter membranous croup and diphtheritic croup will be considered identical to diphtheria by this department, and physicians will be required to report all cases at once by telephone. In case of death, funerals must be private, and take place within twenty-four hours.

Report all cases of whooping-cough.

Physicians are not permitted to remove placards. Whenever the case has recovered (and desquamation ended), so that all danger from contagion or infection has passed, and thorough disinfection has been exercised, this department will cause the removal of the placard upon the request of the attending physician and his statement to the above effect.

Attention is called to Chapter XIV., Section 108, of the City Ordinances, by which pupils are excluded from school as follows :

*Scarlet Fever*—Three weeks from the last case in dwelling.

*Measles*—Two weeks from the last case in dwelling.

*Diphtheria*—One week from the last case in dwelling.

Physicians are requested to be as explicit as possible in filling out birth and death certificates, so that the records of the registrar may contain all necessary data.

The hearty coöperation of the profession is desired, so that the efficiency of this department may be brought to the highest standard.

